# Early public research funding response to COVID-19 pandemic in Brazil

**DOI:** 10.1590/0037-8682-0522-2020

**Published:** 2020-09-21

**Authors:** Kaio Niitsu Campo, Isabella Caroline Pereira Rodrigues, Éder Sócrates Najar Lopes, Laís Pellizzer Gabriel

**Affiliations:** 1 Universidade Federal de Goiás, Faculdade de Ciências e Tecnologia, Aparecida de Goiânia, SP, Brasil.; 2 Universidade Estadual de Campinas, Faculdade de Engenharia Mecânica, Campinas, SP, Brasil.; 3 Universidade Estadual de Campinas, Faculdade de Ciências Aplicadas, Limeira, SP, Brasil.

**Keywords:** Economic development, Health policy, Public policy, Public health systems research, Research and development, Research subsidies, SARS-CoV-2

## Abstract

**INTRODUCTION:**

This study investigated the role of early public research funding regarding the COVID-19 pandemic in Brazil.

**METHODS:**

We examined the budget for research projects relating to the number of cases and deaths and the relationship between each federal unit, gross domestic product (GDP) per capita, and the national GDP per capita.

**RESULTS:**

Using data from the websites of official funding agencies and the Brazilian government, we found that, in the first four months since the first case in Wuhan, China (December 31, 2019), around US$ 38.3 million were directed to public funding for scientific investigations against the COVID-19 pandemic. However, only 11 out of 27 federal units provided funding during the initial stages of the outbreak, and those that did provide financing were not necessarily the units having the most inhabitants, highest GDP, or the greatest number of cases. The areas of research interest were also identified in the funding documents; the most common topic was “diagnosis” and the least common was “equipment for treatment.”

**CONCLUSIONS:**

Brazilian researchers had access to funding opportunities for projects against COVID-19. However, strategies to minimize the economic impacts of COVID-19 are crucial in mitigating or avoiding substantial financial and social shortcomings, particularly in terms of an emerging market such as Brazil.

## INTRODUCTION

The COVID-19 pandemic caused by the severe acute respiratory syndrome coronavirus 2 (SARS-CoV-2) started in Wuhan, China, after many cases with an unknown origin of pneumonia were reported to the World Health Organization (WHO) on December 31, 2019^1^. As of May 31, 2020, more than 6 million confirmed cases and 370,000 related deaths have been reported worldwide^2^. The impact of COVID-19 on human health may range considerably from asymptomatic and mild cases to more critical situations in which severe respiratory failure is observed, primarily in older patients^3^.

On the same day that the WHO declared COVID-19 a global public health emergency (January 30, 2020)^4^, the Brazilian Ministry of Health gathered a working group dedicated to monitoring and taking possible action against the disease^5^. As early as February 3, 2020, the Brazilian government declared a state of emergency^6^; on February 6, 2020, there were nine suspected cases of COVID-19^7^. However, the authorities only confirmed the first case in São Paulo on February 26^8^, which is the biggest, most populous city in Brazil and also the capital of its namesake state. After 20 days, the first death caused by COVID-19 was also confirmed in São Paulo. Since then, the disease spread rapidly throughout the country. According to the official data, on May 31, 2020, there were 514,200 confirmed cases and 29,314 deaths^9^.

Given that there are no vaccines and proven antivirals available yet^10^, one of the main strategies adopted by governments to manage the disease is related to the reduction of transmission rates, which seem to be elevated^11^ ^-^ ^12^. These include social quarantining and lockdowns that, although somewhat controversial, are believed to reduce the need for medical assistance of many people at once^13^ ^-^ ^14^. This is vital because patients needing intensive care can be given more attention and time is gained to increase available resources, such as necessary scientific evidence to fight the disease. In response to the pandemic, the scientific community has published numerous studies covering several areas of expertise. However, many questions and developments remain unanswered or unaccomplished.

Science and technology research in Brazil has been conducted mainly by universities and research institutions, often in partnership with companies. Furthermore, research activities are partly (~50%) funded by the Brazilian government through public funding agencies^15^. Nationally, there are two federal agencies which give funding to researchers: the National Council for Scientific and Technological Development (CNPq)-associated with the Ministry of Science, Technology, Innovation, and Communications-and the Coordination for the Improvement of Higher Education Personnel (CAPES) of the Ministry of Education. Brazil is divided into five macroregions and is further divided into 27 federal units comprised of 26 States and one Federal District (Brasilia, the capital of Brazil). As such, besides the federal foundations, each federal unit has a specific public foundation, known as FAPs (State Research Support Foundations), responsible for providing grants for local research. For example, in São Paulo, the FAP is known as FAPESP (São Paulo Research Support Foundation). Accordingly, each FAP has a distinguished name. Therefore, at least two possible sources for public funding are available for researchers in Brazil, and public funding has been known to have a positive impact on research and development (R&D) production^16^. In this context, this study addresses the early public research funding for scientific investigations specifically against COVID-19 and its consequences in Brazil.

## METHODS

The number of both accumulated and new cases and deaths confirmed in Brazil were obtained on May 1, 2020, from the official national data website (https://covid.saude.gov.br/). The data on the evolution of COVID-19 in Brazil was plotted using a graphic profile. Information related to public research funding for scientific investigations on COVID-19 and its consequences in Brazil from the first four months since the first case (between December 31, 2019, and May 1, 2020) was collected. Public research funding information was collected from the official websites of CAPES and CNPq for national data and FAPs for regional data. Only funding opportunities with official public notice were considered to obtain complete information. Furthermore, they were only contemplated if funded exclusively with national resources. The total amount to be invested by each public research funding agency versus the announcement date was then plotted. All areas of interest for the agencies described in the financing documents were identified. A graph of the total number of occurrences by area was plotted. Finally, the general panorama of Brazilian public research funding during the COVID-19 outbreak was discussed. As reference numbers, data of the federal unit gross domestic product (GDP) and the national GDP were obtained from official data of the Brazilian Institute of Geography and Statistics (2017)^17^. All the funding values presented herein were converted from the Brazilian real to the American dollar on April 30, 2020.

## RESULTS

**FIGURE 1:**
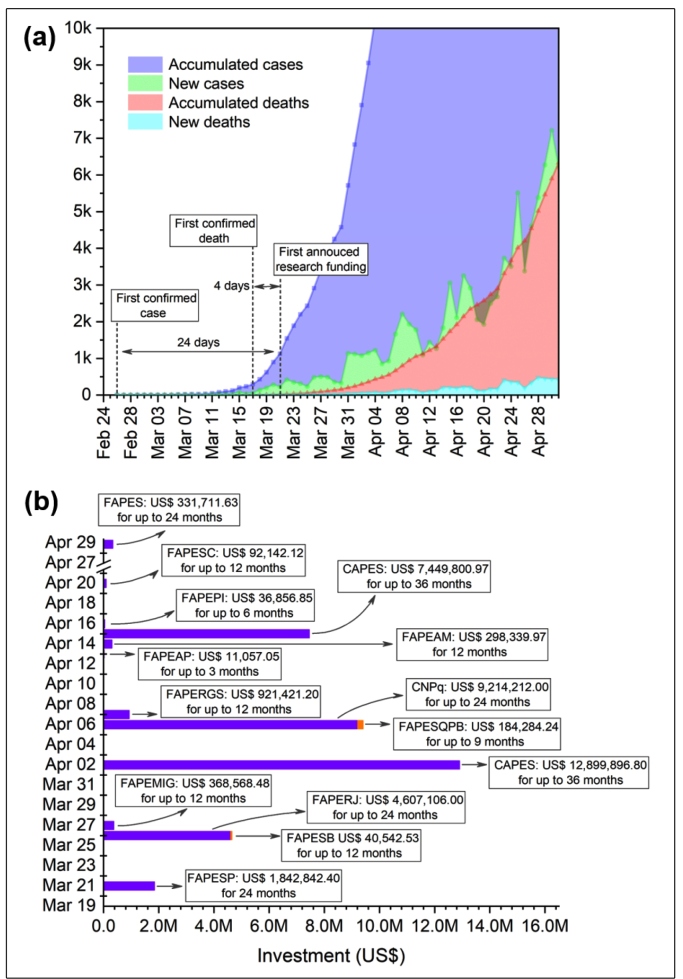
(a) Evolution of confirmed COVID-19 cases and deaths in Brazil (up to May 1, 2020) (b) Early announced public research funding for understanding and fighting COVID-19.

Figure 1(a) depicts the profile of the 91,589 and 6,329 COVID-19 cases and deaths, respectively, from the first case in Brazil up to May 1, 2020. The first research funding focused on COVID-19 was announced on March 21 during the early days of the outbreak, 24 days after the first confirmed case, four days after the first confirmed death, and when the number of accumulated cases and deaths were 1,128 and 18, respectively.

The first funding announcement was made by FAPESP [Figure 1(b)], the foundation (FAP) of São Paulo state, which has the highest GDP in Brazil. The initial budget disclosed by FAPESP for financing COVID-19 related projects was US$ 1,842,842.40. The projects were intended to last for 24 months, but only researchers that already had ongoing projects supported by FAPESP could apply for this rapid funding program. Although not mandatory, the proposal had to be aligned with certain research interests, such as epidemiological features, virus characteristics, diagnostic tests, therapeutic evaluation and development, clinical procedures, and social behavior. It should be noted that FAPESP, in association with the Brazilian Innovation Agency (FINEP), also provided US$ 3,685,684.80 for projects focused on small-sized enterprises to develop technologies for products, services, and processes to fight COVID-19. This was beyond the scope of the current study. 

Five days after the FAPESP announced its funding opportunity [March 26, Figure 1(b)], the Bahia State Research Support Foundation (FAPESB) disclosed a program that offered US$ 40,542.53 in financial support for projects with a maximum duration of 12 months. The proposals had to focus on topics related to the development of clinical protocols, therapeutic guidelines, software, pharmaceuticals, biological products, and equipment for prevention, diagnosis, and therapy. On the same day [Figure 1(b)], the Rio de Janeiro State Research Support Foundation (FAPERJ) opened a program to support COVID-19 research, investing US$ 4,607,106.00. This program was divided into three lines, two were designed to support already granted projects, including projects with companies. The proposals had to address short-to-medium-term solutions against COVID-19. The desired research topics were associated with diagnosis, epidemiology, clinical application, development of innovative technological solutions, and monitoring and forecasting the social impact of measures to deal with the pandemic. In these cases, only projects up to 12 months were accepted. The third program line was devoted to supporting up to six research networks for up to 24 months to deal with epidemic control, diagnostic tests, improvement of level 3 laboratory facilities in Rio de Janeiro State, clinical and epidemiological studies, and solutions involving up to medium-sized businesses. Finally, the last funding program was launched in March by the Minas Gerais State Research Support Foundation (FAPEMIG) which invested US$ 368,568.48 in projects of up to 12 months long.

In April, nine more programs were disclosed [Figure 1(b)], including those of national foundations. On April 6, CNPq provided US$ 9,214,212.00 for proposals concerning the following research topics: COVID-19 treatment, vaccine, diagnosis, pathogenesis and natural history of the disease, disease burden, attention to health, prevention, and control. The deadline execution time was up to 24 months and, in some cases, requests for additional time (12 months) were allowed. On the same day, the Paraíba State Research Support Foundation (FAPESQPB) announced US$ 184,284.24 for proposals up to nine months long. A day later, the Rio Grande do Sul State Research Support Foundation (FAPERGS) announced a grant of US$ 921,421.20 for projects with a maximum duration of 12 months. CAPES launched a program divided into three lines to provide up to US$ 20,349,697.77 to support research activities lasting up to 36 months. This was the largest amount of funding provided in the early stages of the COVID-19 epidemic in Brazil. The first line was launched earlier on April 2 and aimed to fund research proposals related to the epidemic, whereas the second and third lines were disclosed together on April 15 and focused on drugs and immunology, and telemedicine and medical data analysis, respectively. In April, five other state foundations also opened funding opportunities. On April 13, the Amapá State Research Support Foundation (FAPEAP) offered support of US$ 11,057.05 for noticeably short proposals (maximum of three months total duration). On April 14, the Amazonas State Research Support Foundation (FAPEAM) provided US$ 298,339.97 for proposals with a duration of 12 months. On April 16, the Piauí State Research Support Foundation (FAPEPI) called for proposals up to six months long, offering total support of US$ 36,856.85. On April 20, the Santa Catarina State Research Support Foundation (FAPESC) launched a funding opportunity of US$ 92,142.12 for proposals of up to 12 months long. Finally, on April 29, the Espírito Santo State Research Support Foundation (FAPES) announced US$ 331,711.63 for proposals up to 24 months long. These were the main research supports announced within the first months since the first confirmed case of COVID-19 in Brazil.

**FIGURE 2:**
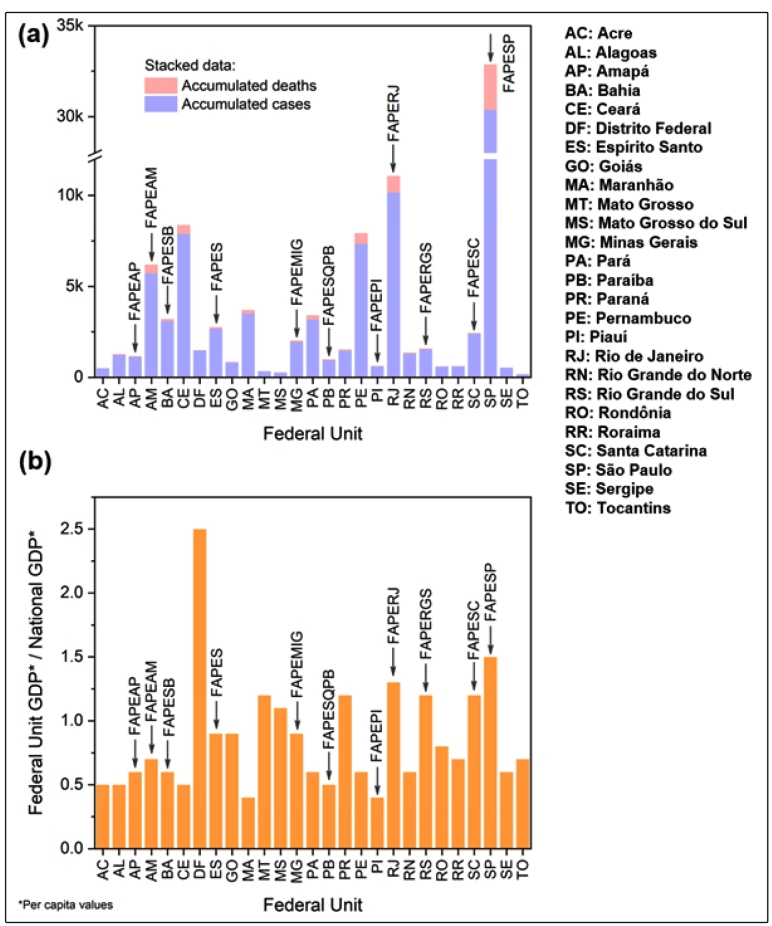
FAPs that launched funding programs and **(a)** accumulated COVID-19 cases and deaths on May 1, 2020, for each Brazilian federal unit, and **(b)** ratio between the national and federal unit GDP per capita.

Total federal agency (CAPES and CNPq) funding amounted to US$ 29,563,909.77, representing around 77% of the total amount for early COVID-19 research funding. Funding from FAPs was significantly lower; only 11 of 27 FAPs provided early funding opportunities, US$ 8,734,872.48 in total, for scientific research in response to the COVID-19 pandemic. Figure 2(a) shows the relationship between the accumulated deaths and cases, as of May 1, and the FAPs that have disclosed funding programs. Most FAPs of federal units with few cases and deaths did not initially invest in research, while the FAPs of federal units with the most cases and deaths, São Paulo (SP) and Rio de Janeiro (RJ), did. One should note that some FAPs of federal units with comparatively high incidences of COVID-19, such as Ceará (CE) and Pernambuco (PE), did not follow the regional funding policy. However, in fact, both FAPs announced funding opportunities, but they did not meet the criteria to be included here. Figure 2(b) presents the ratio between the GDP per capita of each federal unit and the national GDP per capita. This shows that the financial strength of the federal unit was not decisive in opening funding initiatives, since FAPEPI from Piauí (PI), which has one of the lowest GDPs per capita in Brazil, was one of the FAPs that supported research on COVID-19. PI was also one of the federal units with the lowest accumulated deaths and cases as of May 1 [Figure 2(a)], suggesting that the number of cases and deaths did not always play a role in stimulating the emergency funding programs.

**FIGURE 3:**
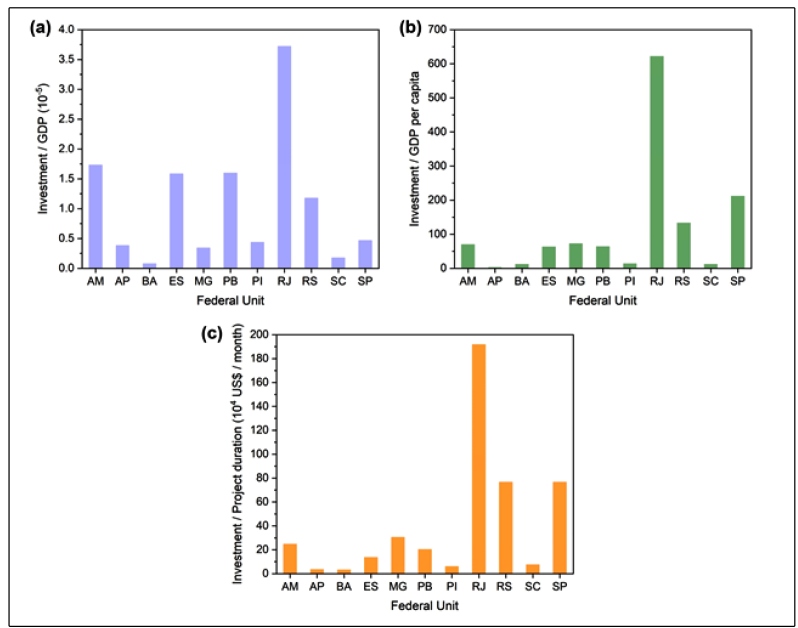
Investment of FAPs for research proposals against COVID-19 as a function of **(a)** federal unit GDP, **(b)** federal unit GDP per capita, and (c) maximum project duration.

Figure 3 shows a more detailed view of the investments made by the FAPs. Investment as a function of the federal unit GDP is presented in Figure 3(a). FAPERJ from RJ was the FAP that provided both the highest total investment and investment relative to the federal unit GDP. Although SP is the federal unit with the highest GDP, the proportion of investment in COVID-19 research was less than that of poorer federal units, such as FAPESQPB from Paraíba (PB). However, the investment provided by FAPESP (SP) was only lower than that of FAPERJ (RJ) when considered a function of the federal unit GDP per capita [Figure 3(b)]. Another means of investigating these funding opportunities is by considering the investment as a function of the project duration [Figure 3(c)]. Again, FAPERJ (RJ) had the highest monthly investment, followed by FAPESP (SP) and FAPERGS (Rio Grande do Sul). Although FAPESQPB offered a comparatively elevated investment relative to its GDP, when the monthly investment was considered, it was much less than that of RJ.

**FIGURE 4:**
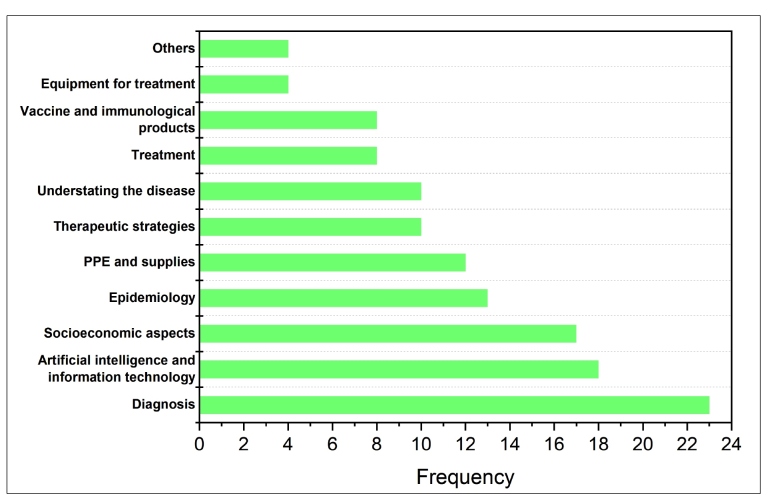
Areas of research interest described in the funding documents.

The areas of research interest of each public funding agency highlighted in the funding documents are summarized in Figure 4. They are discussed here from most to least frequently researched. The most frequently researched topic was “diagnosis,” which reflects the search for rapid and reliable means to determine whether a person was infected with COVID-19. This is vital to taking appropriate action against the spread of the disease and to manage infected individuals^18^. Meanwhile, “artificial intelligence and information technology” can be discussed mainly in terms of artificial intelligence (AI), which has been widely investigated as an efficient tool against COVID-19. Vaishya et al. listed the main possible applications of AI in the current pandemic^19^, including providing support for early diagnosis, monitoring treatment and virus spread, identifying “hot-spots,” predicting disease evolution, developing drugs and vaccines, and assisting healthcare workers^19^. “Socioeconomic aspects,” which comprises actions to avoid or mitigate social and economic impacts resulting from the measures taken to combat and control the COVID-19 outbreak, were also widely researched. The importance of this aspect is highlighted by FAPEAP (Amapá) providing financial support, albeit considerably miniscule, exclusively for proposals aiming to reduce regional economic adversities [Figure 1(b)]. 

Meanwhile, “epidemiology” investigated the damage caused by COVID-19 on the health of the Brazilian population to understand the potential impact of COVID-19 control measures, monitor the disease evolution, and developing strategies to end social distancing. “Personal protective equipment (PPE) and supplies for healthcare workers” were related to disease spread control. Given that the international supply chain has been affected by the pandemic^20^, the shortage of materials is a problem not just in Brazil but worldwide. Therefore, the development of innovative ideas to overcome the shortages for all kinds of suppliers is crucial. “Therapeutic strategies” were of interest due to the development of clinical protocols and therapeutic guidelines, while “understanding the disease” referred to genetic aspects of the virus and pathophysiology and clinical aspects of the disease. As for “treatment,” there has been interest in studying therapeutic alternatives for COVID-19, with focus on antiviral drugs. Along with “treatment,” “vaccines and immunobiological products” have also been researched. Worldwide, significant efforts have been made to develop effective vaccines and drugs against COVID-19^21^ ^-^ ^22^. Finally, the research interest topic that occurred the least was “equipment for treatment,” which comprises the development of low-cost pulmonary ventilators and equipment for treatment and isolation of patients. “Others” refers to studies in different fields that could not be fit into the other categories and were only mentioned once, such as the development of robotics for hospital applications.

## DISCUSSION

The present analysis focused on the response of public research funding agencies in Brazil to the early COVID-19 pandemic period. Science and technology research in Brazil has mainly been conducted by universities and research institutions, funded by the Brazilian government through public funding agencies. These funds are granted by federal agencies and FAPs, nationally and regionally, respectively. In the past few years, the total annual public research funding in Brazil has been around US$ 10 billion^15^. Over the first four months since the outbreak began in China, the public funding agencies of the eleven federal units and two federal agencies provided US$ 38,298,782.25 for scientific investigations. Although this amount is much lower than the total annual amount, it should be considered that it has been provided on an emergency basis, besides the fact that it may increase over the year. 

Brazil is a large country, geographically divided into 27 federal units (26 states and one federal district), but, according to the number of COVID-19 cases and GDP per capita, only 11 of these 27 units provided the US$ 8,734,872.48 in early funding opportunities for scientific researches. The number of cases and deaths did not always play a role in stimulating the emergency funding programs. The COVID-19 pandemic promoted a large worldwide commotion, probably encouraging some FAPs to provide research funding even if the number of cases were not high regionally. Furthermore, early actions could help to find better ways to manage and avoid possible future problems. A more detailed analysis investigated FAP investment as a function of the federal unit GDP. However, the results showed that the federal GDP did not affect the amount spent on research. Finally, the areas of research interest identified in the funding documents were classified. The topic with the highest and least occurrence were “diagnosis” and “equipment for treatment,” respectively. 

The main limitations of this study are related to the fact that only public notices with complete information (such as funding amount, project duration, and areas of research interest) were analyzed. Furthermore, only funding opportunities from government agencies using exclusively national resources were considered. This means that more resources may have been allocated for financing COVID-19 related projects during the analyzed period. Another point to be acknowledged is that the funding documents are not uniform in their contents, that is, the programs were not developed in the same way. For instance, two different programs were separately announced by FAPESP (SP), one developed for financing projects in universities and research institutions, and another for enterprises. On the other hand, although this study did not focus on projects to be exclusively performed by companies, it was not possible to make a clear division between the previous target audiences when analyzing the program disclosed by FAPERJ (RJ). This discrepancy may have contributed somewhat to the results showing the much higher investment amount provided by FAPERJ (RJ) in comparison with FAPESP (SP). 

This work showed that Brazilian researchers had access to funding opportunities for projects aiming to address issues related to COVID-19 since the beginning of the outbreak in Brazil. Nonetheless, future opportunities are at risk because of the economic impacts derived from the measures taken to control the disease. Accordingly, strategies to minimize the economic impacts of COVID-19 on public research and the public health system are vital in mitigating or avoiding substantial financial and social shortcomings, particularly from the perspective of an emerging market such as Brazil. Science and technology innovation and development in Brazil, especially during emergencies, may be conducted with cooperation between universities and industries. It has been shown that this may be beneficial and facilitate problem-solving^23^, and, in the long-term, the government, can provide a tax incentive to industries that worked with and continue to promote cooperation with universities. In the short-term, the Brazilian government and public research funding agencies will have to develop innovative solutions to overcome an economic crisis that is likely to have a strong effect on scientific activities, such as massive drops in funding, scientific publications, patents, and qualified human resources.
